# A new species of *Stenobiella* Tillyard (Neuroptera, Berothidae) from Australia

**DOI:** 10.3897/zookeys.64.403

**Published:** 2010-10-22

**Authors:** Shaun L. Winterton

**Affiliations:** California State Collection of Arthropods, California Department of Food & Agriculture, 3294 Meadowview Rd. Sacramento, CA, USA 95832-1148

**Keywords:** Berothidae, Neuroptera, lacewing

## Abstract

Stenobiella variola **sp. n.**, a new species of beaded lacewing (Neuroptera: Berothidae), is described and figured from south-eastern Australia. A preliminary key to Stenobiella species is presented.

## Introduction

Beaded lacewings (Berothidae) are a small family of Neuroptera comprising approximately 100 species occurring throughout most biogeographical regions. Members of the family are recognised by elongation of the pronotum, female usually with hypocaudae and substantial cubital veins in both wings. The larvae are associated with termites, and undergo a degree of hypermetamorphosis during development (Brushwein 1987).

Four subfamilies of Berothidae are recognised: Rhachiberothinae, Cyrenoberothinae, Berothinae and Nosybinae (Aspöck 1986; MacLeod and Adams 1967; New 1989). Rhachiberothinae have been considered by some authors as a separate family (Aspöck and Mansell 1994) or as a subfamily of Mantispidae (Willmann 1990). A fifth subfamily, Nyrminae, was erected by Aspöck (1989) based on a highly autapomorphic species (Nyrma kervillea Navás) previously placed in Hemerobiidae. Penny and Winterton (2007) recently rediscovered the enigmatic genus Ormiscocerus Blanchard from Chile and placed it in Cyrenoberothinae based on various wing and genitalic characteristics; a placement also supported in phylogenetic analyses by Winterton et al. (2010). Like Nyrma Navás, Ormiscocerus was also previously placed in Hemerobiidae and the wing venation of both species show numerous similarities, indicating that Nyrma should be placed in Cyrenoberothinae rather than as a separate subfamily. In a cladistic analysis of Berothidae using morphology Aspöck and Nemeschkal (1998) proposed a major reordering of the internal hierarchy and classification of the family with five subfamilies (Cyrenoberothinae, Trichomatinae, Protobiellinae, Nosybinae and Berothinae).

Stenobiella Tillyard (Berothinae) is an endemic Australian genus originally described based on two species (Stenobiella hirsutissima Tillyard and Stenobiella gallardi Tillyard) from Queensland and New South Wales (Tillyard 1916). Kimmins (1930) described a third species of (Stenobiella pulla Kimmins) from the Northern Territory and Aspöck and Aspöck (1984) subsequently described seven new species, bringing the total number of species to 10. An eleventh species is described and figured herein (Stenobiella variola sp. n.) from western New South Wales. A preliminary key to species is presented.

## Methods

Genitalia were macerated in 10% KOH at room temperature for one day to remove soft tissue, then rinsed in distilled water and dilute acetic acid and dissected in 80% ethanol. Preparations were then placed into glycerine, with images made with the aid of a digital camera mounted on a stereomicroscope. Genitalia preparations were placed in glycerine in a genitalia vial mounted on the pin beneath the specimen. Terminology follows MacLeod and Adams (1967) and Aspöck and Aspöck (1984). Specimen images were taken using a digital camera with a series of images montaged using Helicon Focus (©HeliconSoft) and links provided to Morphbank for high-resolution images. All new nomenclatural acts and literature are registered in Zoobank^1^ as per the recent proposed amendment to the International Code of Zoological nomenclature for a universal register for animal names (Polaszek et al., 2005a, b; Pyle et al., 2008; ICZN 2008).

http://www.zoobank.org/

## Taxonomy

### Stenobiella variola sp. n.

urn:lsid:zoobank.org:act:1F7A88BE-C893-4737-A852-6DD0E569937B

Figs 1 [Fig F2] 3 

#### Holotype

male, AUSTRALIA: New South Wales: Tintinallogy Station, 15.i.2010, light sheet, -31.9994°, 143.01706°, S.L. Winterton & N.B. Hardy, light sheet (Australian National Insect Collection).

#### Paratypes.

AUSTRALIA: New South Wales: 2 males, 1 female, same data as holotype (California Academy of Science Collection).

**Figure 1.**
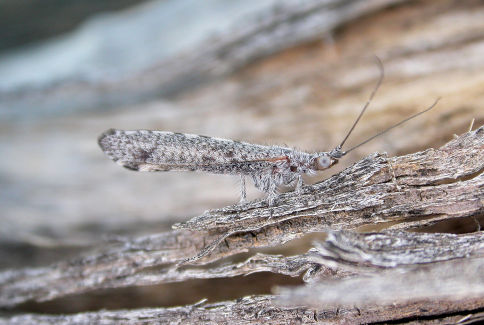
Stenobiella variola sp. n. Female habitus. Photo credit: Shaun L. Winterton.

#### Diagnosis.

Distinctively contrasted variegated wing pattern; numerous white non-tapered setae on wings and body, pale patch basally in pterostigma, darker distally; dark scale-like setae absent from wings and abdomen; dark, elongate setae absent from mid coxa; single R1-Rs cross-vein; dark, elongate setae along entire posterior margin of both wings; female hypocaudae well developed; male paramere-mediuncus complex relatively large.

#### Description.

Body length = 5.0–6.0 mm (male), 6.1 mm (female). *Head*. Black to light brown; anterior tentorial pits distinct; clypeus with dark band and minute pale pubescence; vertex irregularly covered with elongate, non-tapered white setae, multidirectional and partially appressed; raised lateral tubercle with elongate white setae admixed with several longer and more tapered black setae; antenna dark brown to black, scape covered with elongate white setae admixed with dark setae; pedicel with ring of dark setae basally, closely approximating a distal ring of white setae; 51 flagellomeres covered with fine dark setae; mouthparts brown with sparse black setae.

**Figure 2. F2:**
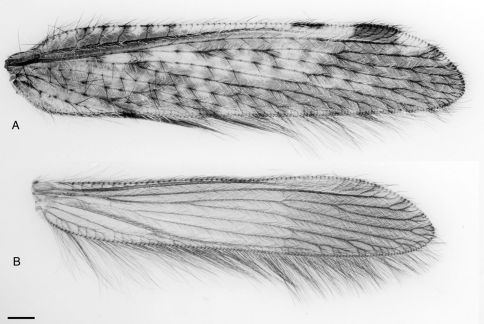
Stenobiella variola sp. n., **A** forewing **B** hindwing. Scale line = 0.5 mm.

*Thorax*. Pronotum wider than long, dark brown; two latitudinal depressions extending from midline, each with white setae along length; white setae along midline and around margin; admixed with slightly longer and tapered black setae along lateral and anterior margins; mesonotum light brown, blackish posterolaterally, white setae anteromedially in ‘V’ pattern, admixed with patch of dark setae; dark area glabrous; metathorax light brown with dark patches laterally, posterior portio with tapered pale setae; pleuron with extensive white, non-tapered setae. *Wing* (Figure 2). Forewing length = 6.1 mm. Hind wing length = 5.2 mm. Forewing hyaline with extensive infuscate mottling; venation brown and tan mottled, numerous dark tapered macrosetae along wing veins with infuscate area around base of each seta; rows of white non-tapered setae extensive along all wing veins, admixed with dark, non-tapered setae, distribution of white and dark non-tapered setae relative to surrounding infuscation (i.e. more white setae in hyaline areas); costal and subcostal areas with extensive infuscation, white areas along costal margin with dark mark basad of pterostigma; pterostigma dark with white either side; dark, elongate along entire posterior margin of wing; single cross-vein between R_1_ and anterior trace of Rs; 4–5 gradate series cross-veins; distal CuA-MP cross-vein perpendicular to CuA and originating on anterior branch of distal CuA fork; hindwing hyaline; venation light brown to yellow; macrosetae absent, extensive fine tapered setae on all veins, more numerous in distal area of wing and much longer along entire posterior margin of wing. *Legs*. Uniform dark brown with extensive covering of elongate, white setae; setae shorter and darker on tarsomeres.

*Abdomen*. Uniform brown to light brown; extensive pile of dark setae admixed with white setae, denser on sternites; stripe of white, non-tapered setae laterally from segment one to terminalia

**Figure 3.**
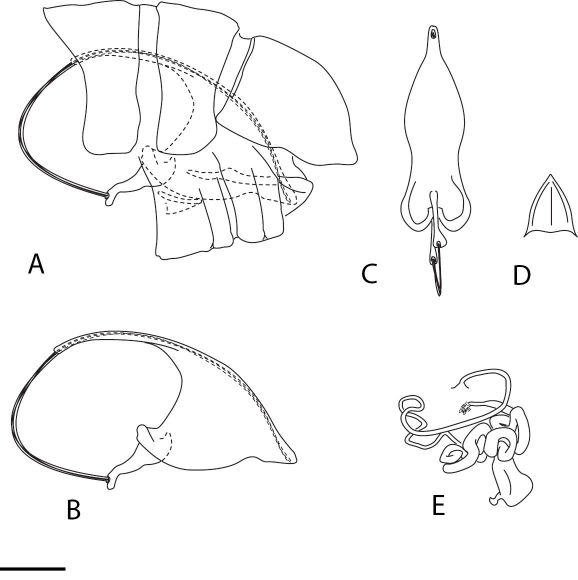
Stenobiella variola sp. n., Male genitalia: **A** Genital segments, lateral **B** paramere-mediuncus complex, lateral **C** same, ventral **D** hypandrium internum, ventral. Female genitalia: **E** spermatheca, ventral. Scale line = 0.2 mm.

*Male genitalia* (Fig. 3A–D). Tergite 9 + ectoproct rounded posteriorly, slightly acuminate distally; paramere-mediuncus complex very large with well sclerotised guide; hypandrium internum triangular.

*Female genitalia* (Fig. 3E). Hypocaudae well developed, elongate; spermatheca large and highly convoluted in shape.

#### Etymology.

The specific epithet is derived from Latin, *variola*; spotted, mottled.

#### Comments.

Stenobiella variola sp. n. is a distinctive species based on wing mottling and extensive wing and body covering of white, non-tapered setae. The male genitalia are similar in structure to Stenobiella theischingerorum Aspöck & Aspöck, to which Stenobiella variola sp. n. appears to be closely related. No key to species of Stenobiella exists. The following key is based largely on the published descriptions by Aspöck and Aspöck (1984) and examination of additional non-type material in various collections; considering the high likelihood of new species being collected, it should be considered preliminary only. Both sexes are required for the key to work most effectively for some species.

#### Key to Stenobiella species

**Table:** 

1.	Wing mostly or completely pale; relatively large species (ca. 8.0–10.0 mm forewing length); hypocauda present as a relatively short, blunt process	2
–	Wing dark infuscate to stark maculate; size variable, but usually less than 9.0 mm forewing length; hypocauda as elongate process (rarely greatly reduced to a knob)	3
2.	Wing with costal field dark	Stenobiella kaikai Aspöck & Aspöck
–	Wing uniformly pale	Stenobiella arrunja Aspöck & Aspöck
3.	Male paramere-mediuncus complex relatively small (cf. Aspöck and Aspöck 1984: figs 21, 31)	4
–	Male paramere-mediuncus complex relatively large (Fig. 3A–B)	5
4.	Black scale-like setae present on both wings	Stenobiella cardaleae Aspöck & Aspöck
–	Black scale-like setae absent on both wings	Stenobiella muellerorum Aspöck & Aspöck
5.	Female hypocauda knob-like; spermatheca large; paramere-mediuncus complex shape as in Aspöck and Aspöck (1984: fig. 11)	Stenobiella pindana Aspöck & Aspöck
–	Female hypocauda elongate; spermatheca smaller; paramere-mediuncus complex shape as in Figure 3 A–C	6
6.	Scales on forewing present	Stenobiella moma Aspöck & Aspöck
–	Scales on forewing absent	7
7.	Wing largely uniform infuscate but not distinctly maculate	Stenobiella pulla Kimmins, Stenobiella gallardi Tillyard, Stenobiella hirsutissima Tillyard
–	Wing distinctly maculate	8
8.	Costal field with alternating dark and pale regions to forewing tip (cf. Aspöck and Aspöck 1984: fig. 46–47); hypandrium apex relatively tapered; paramere-mediuncus shape as in Aspöck and Aspöck 1984: figs 3–4 (Northern Territory)	Stenobiella theischingerorum Aspöck & Aspöck
–	Costal field with alternating dark and pale regions to forewing tip but with distinct pale area midway (Fig. 2A); hypandrium apex not as acutely tapered; paramere-mediuncus shape as in Fig. 3A–C (New South Wales)	Stenobiella variola sp. n.

## Supplementary Material

XML Treatment for Stenobiella variola

## References

[B1] Aspöck U Aspöck H (1984) Die Berothiden Australiens I: Neue spezies des genus *Stenobiella* Tillyard (Neuropteroidea: Planipennia: Berothidae). Zeitschrift der Arbeitsgemeinschaft Österr. Entomolgen 36:17-32

[B2] Aspöck U (1986) The present state of knowledge of the family Berothidae (Neuropteroidea: Planipennia). In: Gepp J Aspöck H Hölzel H (Eds) Recent Research in Neuropterology. Proceedings of the 2nd International Symposium on Neuropterology (21–23 August 1984, Hamburg, Germany; held in association with the XVII International Congress of Entomology), Graz, Austria, 87–101

[B3] Aspöck U Nemeschkal HL (1998) A cladistic analysis of the Berothidae (Neuroptera). Acta Zoologica Fennica 209:45-63

[B4] Aspöck U (1989) *Nyrma kervillea* Navás – eine Berothide! (Neuropteroidea: Plannipennia). Zeitschrift der Arbeitsgemeinschaft Österr. Entomolgen 41:19-24

[B5] Aspock U Mansell MW (1994) A Revision of the Family Rhachiberothidae Tjeder, 1959, stat. n. (Neuroptera). Systematic Entomology 19:181-206

[B6] Brushwein JR (1987) Biomonics of *Lomamyia hamata* (Neuroptera: Berothidae). Annals of the Entomological Society of America 80:671-679

[B7] International Commission on Zoological Nomenclature (2008) Proposed amendment of the International Code of Zoological Nomenclature to expand and refine methods of publication. Zootaxa 1908:57-67

[B8] Kimmins DE (1930) A new Australian berothid (Neuroptera). Entomologist’s Monthly Magazine 66:162-163

[B9] MacLeod EG Adams PA (1967) [1968] A review of the taxonomy and morphology of the Berothidae, with the description of a new subfamily from Chile (Neuroptera). Psyche 74:237-265

[B10] New TR (1989) Planipennia. Lacewings. Handbuch der Zoologie (Berlin) 4:1-132

[B11] Penny ND Winterton SL (2007) Rediscovery of the unusual genus *Ormiscocerus* (Neuroptera: Berothidae: Cyrenoberothinae). Proceedings of the California Academy of Sciences 58:1-6

[B12] Polaszek A Agosti D Alonso-Zarazaga M Beccaloni G de Place Bjørn P Bouchet P Brothers DJ Earl of Cranbrook Evenhuis NL Godfray HCJ Johnson NF Krell FT Lipscomb D Lyal CHC Mace GM Mawatari SF Miller SE Minelli A Morris S Ng PKL Patterson DJ Pyle RL Robinson N Rogo L Taverne J Thompson FC van Tol J Wheeler QD Wilson EO (2005a)Commentary: A universal register for animal names. Nature 437: 477 10.1038/437477a 16177765

[B13] Polaszek A Alonso-Zarazaga M Bouchet P Brothers DJ Evenhuis NL Krell FT Lyal CHC Minelli A Pyle RL Robinson N Thompson FC van Tol J (2005b) ZooBank: the open-access register for zoological taxonomy: technical discussion paper. Bulletin of Zoological Nomenclature 62:210-220

[B14] Pyle RL Earle JL Greene BD (2008) Five new species of the damselfish genus *Chromis* (Perciformes: Labroidei: Pomacentridae) from deep coral reefs in the tropical western Pacific. Zootaxa 1671:3-31

[B15] Tillyard RJ (1916) Studies in Australian Neuroptera. No. iv. The families Ithonidae, Hemerobiidae, Sisyridae, Berothidae, and the new family Trichomatidae; with a discussion of their characters and relationships, and descriptions of new and little-known genera and species. Proceedings of the Linnean Society of New South Wales 41:269-332

[B16] Willmann R (1990) The Phylogenetic position of the Rhachiberothinae and the basal sister-group relationships within the Mantispidae (Neuroptera). Systematic Entomology 15:253-265

[B17] Winterton SL Hardy NB Wiegmann BM (2010) On wings of lace: phylogeny and Bayesian divergence time estimates of Neuropterida (Insecta) based on morphological and molecular data. Systematic Entomology 35:349-378

